# Neuropsychological and Academic Performance in Colombian Children with Attention-Deficit Hyperactivity Disorder: A Comparative Study with a Control Group

**DOI:** 10.3390/children12050561

**Published:** 2025-04-26

**Authors:** Daniel Landínez-Martínez, Diana Montoya-Londoño, Lorena Aguirre-Aldana, Carmen Dussán-Lubert, Carolina Robledo-Castro, Antonio Partida-Gutierrez de Blume

**Affiliations:** 1 Social Sciences, Health and Welfare Faculty, Universidad Católica Luis Amigó, Manizales 170001, Colombia; 2 Faculty of Health Sciences, Universidad de Manizales, Manizales 170001, Colombia; 3 Educational Studies Department, Faculty of Arts and Humanities, Universidad de Caldas, Manizales 170001, Colombia; diana.montoya@ucaldas.edu.co; 4 Faculty of Social and Human Sciences, Universidad de Manizales, Manizales 170001, Colombia; laguirrea@umanizales.edu.co; 5 Department of Mathematics, Faculty of Exact and Natural Sciences, Universidad de Caldas, Manizales 170001, Colombia; carmen.dussan@ucaldas.edu.co; 6 Department of Pedagogy and Technological Mediations of the Distance Education Institute (IDEAD), Universidad del Tolima, Ibagué 730004, Colombia; crobledoc@ut.edu.co; 7 Department of Curriculum, Foundations, and Reading, Georgia Southern University, Statesboro, GA 30458, USA; agutierrez@georgiasouthern.edu

**Keywords:** attention-deficit/hyperactivity disorder, neurocognitive performance, executive functioning, metalinguistic skills, memory, attention, language

## Abstract

**Objective:** This study aimed to determine the effect of ADHD on the neuropsychological and academic performance of a sample of Colombian children in primary and secondary education compared to a control group. **Method:** Quasi-experimental research design involving a sample of 194 children from Manizales, of whom 97 were diagnosed with ADHD and 97 were typically developing children. The study utilized tasks from the Child Neuropsychological Assessment (ENI) protocol to assess academic and neuropsychological performance. **Results:** Children with ADHD exhibited lower cognitive, linguistic, and attentional performance with greater variability than their neurotypical peers. They showed deficits in IQ, metalinguistic skills, reading, writing, memory, attention, and executive function, with increased errors and heterogeneity across tasks. **Conclusions:** For future research, it is necessary to address ADHD through mixed-methods studies that enrich quantitative findings with the lived experiences of children and families affected by ADHD. Additionally, further exploration is needed regarding functional impairment assessment in the Colombian and broader Ibero-American context, including its correlation with later academic performance in higher education.

## 1. Introduction

Attention-deficit/hyperactivity disorder (ADHD) is a childhood-onset neurodevelopmental disorder characterized by a persistent pattern of inattention and/or hyperactivity–impulsivity that significantly interferes with functional development. According to current diagnostic criteria, the presence of six or more symptoms of inattention and/or hyperactivity–impulsivity is required for a diagnosis. These symptoms must persist for a minimum of six months, be developmentally inappropriate, and exert a direct negative impact on academic, social, and, in some cases, occupational functioning [1,2,3,4]. Recent studies suggest that ADHD persists into adulthood in approximately 6.7% of cases, affecting an estimated 366.33 million adults worldwide [5].

Prevalence estimates for ADHD vary considerably, with rates ranging from 3.4% to 20% depending on diagnostic criteria, population characteristics, and methodological differences across studies [6,7,8,9]. The American Psychiatric Association (2022) classifies ADHD into three primary presentations: predominantly inattentive, predominantly hyperactive–impulsive, and combined. Meta-analytic evidence indicates that the inattentive presentation accounts for approximately 33.2% of cases, the hyperactive–impulsive for 30.3%, and the combined type for 31.4% [10,11,12]. Additionally, research highlights that ADHD prevalence and symptomatology differ across ethnic, geographic, economic, and educational contexts [6,13,14,15]. Systematic reviews incorporating data from diverse regions—including China, India, Africa, the United States, and Ibero-America—support this variability, reporting global prevalence estimates within the aforementioned range [16,17,18]. These findings underscore the importance of continued research to refine theoretical models, enhance diagnostic precision, and develop contextually relevant interventions, particularly in light of ADHD’s significant implications for academic performance, psychosocial functioning, and overall quality of life [19,20,21,22,23,24].

Numerous studies comparing the neuropsychological and academic performance of children with ADHD to that of typically developing peers have consistently reported deficits in working memory, attention, executive functions, and reading comprehension [13,20,25,26,27,28]. However, given the disruptions caused by the COVID-19 pandemic, it is essential to reassess these cognitive and academic profiles in the post-pandemic context. Emerging research suggests that children with ADHD now exhibit even greater difficulties in sustaining attention, engaging with academic tasks, and regulating their learning behaviors. The shift toward remote and digital learning environments may have exacerbated these challenges, placing children with ADHD at an even greater disadvantage compared to their peers [29,30,31].

Additionally, post-pandemic educational reforms have placed increased emphasis on autonomous and independent learning, a requirement that may be particularly challenging for children with ADHD. Given that executive function impairments are a core feature of ADHD, these increased demands may further impact their academic performance and learning outcomes [32,33].

In light of these evolving challenges, it is crucial to update our understanding of the neuropsychological and academic profiles of children with ADHD. Such knowledge will inform the development of tailored psychological and educational interventions aimed at supporting their cognitive and academic growth.

Previous research has documented considerable variability in the cognitive and academic profiles associated with ADHD across diverse sociocultural contexts. Table 1 provides a summary of key studies examining these differences in comparison to control groups. This body of evidence underscores the heterogeneous neuropsychological profile of ADHD, reinforcing the understanding that it comprises multiple manifestations with distinct cognitive and functional implications. Studies conducted in Spain, Colombia, the Netherlands, Belgium, and the Dominican Republic consistently report that children with ADHD exhibit marked deficits in executive functioning, working memory, attentional control, and metalinguistic abilities relative to neurotypical peers. Furthermore, ADHD subtypes demonstrate distinct patterns in inhibitory control, processing speed, and attentional regulation, highlighting the importance of subtype-specific diagnostic approaches and intervention strategies.

Beyond core cognitive impairments, the severity of attentional and inhibitory deficits emerges as a primary predictor of both symptom severity and functional impairment [34]. The frequent co-occurrence of learning disorders [28] and overlapping neurodevelopmental conditions—such as ADHD and dyslexia [27]—further supports the need for comprehensive neuropsychological assessments. Collectively, these findings call for a more nuanced conceptualization of ADHD and emphasize the relevance of individualized, evidence-based interventions that account for cognitive diversity and its functional impact.

**Table 1:** Research background on the neuropsychological and academic profiles of children with ADHD.

Method	Country	Findings	Study
Sample: Children aged from 6 to 13 years diagnosed with ADHD. Aim: To analyze the cognitive profiles of children with ADHD and to examine the differences between them.	Spain	In the evaluation of the two neuropsychological profiles, no significant differences were found in the index of verbal comprehension, perceptual reasoning, and total intelligence quotient/general ability index. However, the ADHD group showed lower scores than the control group in working memory. Regarding the processing speed index, the control group exhibited lower performance than the group of children with ADHD.	[35]
Sample: Children aged 5 to 15 diagnosed with ADHD. Objective: To identify differences in neuropsychological performance between a group of children diagnosed with combined-type and inattentive-type ADHD and a group of typically developing children.	Colombia	Differences were found between the case and control groups in the cognitive processes of memory, attention, and language, with lower scores in these areas for the case group compared to the controls. However, no significant differences were observed in the evaluation of executive functions. In the memory process, the case group demonstrated significantly lower average performance than the control group in auditory–verbal memory tasks, particularly in encoding and retrieval. Regarding attention, the case group exhibited lower average scores than the control group in auditory attention tasks, such as backward digit span, along with a higher number of errors and omissions. In terms of language, the case group performed worse than the control group in tasks assessing instruction-following and metalinguistic skills.	[13]
Sample: Children aged 6 to 16 years diagnosed with ADHD. Objective: To compare the neurobiological functioning of children and adolescents with ASD and ADHD in the city of Manizales.	Colombia	In the comparison of the three subgroups—ADHD, Asperger’s, and control—the ADHD group demonstrated lower performance in visuo-constructional and memory tasks, particularly in spontaneous recall and cued recall tests. Regarding executive functions, the ADHD group obtained the lowest average scores in backward and forward digit span tasks, as well as in the number of categories completed. In terms of language performance, the ADHD group showed the lowest scores in verbal fluency and instruction-following tasks.	[36]
Sample: Children aged 5 to 14 years with ADHD and 97 control subjects. Objective: To examine metalinguistic skills and reading processes in children diagnosed with ADHD, compared to a matched control group.	Colombia	Children diagnosed with ADHD demonstrated significantly lower performance than their neurotypical peers across all assessed metalinguistic and reading tasks, with the exception of spelling accuracy and silent reading comprehension, where no statistically significant differences were observed.	[20]
Sample: Children aged 8 to 16 years with ADHD. Objective: To establish the relationship between two of the main cognitive deficits in ADHD (attention and inhibitory control), symptomatology (inattention and hyperactivity/impulsivity), and functional impact in patients diagnosed with ADHD without comorbid disorders.	Spain	The results indicated that greater deficits in cognitive functioning (attention and inhibitory control) predicted higher ADHD symptom severity (inattention and hyperactivity/impulsivity). Regarding the relationship between neuropsychological functioning and functional impact, the data suggested that greater attentional and inhibitory deficits predicted greater functional impairment, but only through the mediation of symptom severity.	[34]
Sample: Children aged 6 to 14 years diagnosed with ADHD. Objective: To compare the neuropsychological performance characteristics of a sample of children with combined-type ADHD (ADHD-C), inattentive-type ADHD (ADHD-I), and a control group from the city of Manizales, Colombia.	Colombia	Differences were found in performance on visual attention tasks, with lower mean scores for the ADHD-C group compared to the ADHD-I group. Additionally, the ADHD-I group showed lower mean scores in metalinguistic skills (sound counting) compared to the control group. No significant differences were found in other measures included in the evaluation, such as intellectual capacity, memory, and executive functions.	[37]
Sample: Children aged 10 to 14 years diagnosed with ADHD. Objective: To analyze the relationship between the neuropsychological profile and the level of emotional intelligence in fifth-grade children with suspected ADHD.	Dominican Republic	In the evaluation of the neuropsychological profile, the assessed group demonstrated a low-average performance in intellectual capacity measures. Additionally, cognitive measures derived from the DNI-Luria battery indicated low performance in tasks related to visual perception, spatial orientation, receptive speech, conceptual activity, immediate memory, logical memory, and attentional control. No association was found between intelligence quotient, neuropsychological profile, and emotional intelligence measures.	[38]
Sample: Children aged 5 to 6 years, with and without ADHD. Objective: To compare the cognitive profile of preschool children at risk of dyslexia with the cognitive profile of children at risk of both dyslexia and coexisting ADHD.	Belgium	When comparing the group of children at risk for dyslexia with those at risk for both dyslexia and coexisting ADHD, no significant differences were found in most cognitive measures, except for executive functioning, where the dyslexia-only group performed better than the dyslexia–ADHD comorbidity group. The results indicated that the control group generally outperformed both risk groups across all evaluated measures, including phonological processing, executive functioning, receptive vocabulary, and processing speed, except for cognitive flexibility and delay of gratification.	[27]
Sample: Children with ADHD aged 6 to 15 years and 24 control children aged 7 to 15 years. Objective: To describe the neuropsychological profile of patients with attention-deficit/hyperactivity disorder (ADHD) and its impact on executive functions and academic performance.	Spain	Children and adolescents with ADHD showed significantly lower scores than the neurotypical control group in all cognitive measures (motor functions, verbal abilities, abstract reasoning, linguistic, memory, attentional, and executive functions, as well as academic skills), except for perceptual abilities. More than half of the evaluated ADHD sample had a comorbid learning disorder.	[28]

Note: The studies summarized in this table were identified through systematic searches of PubMed, Scopus, and Web of Science databases, covering the period from 2011 to 2024.

Given the heterogeneous cognitive and academic profiles associated with ADHD, particularly across diverse sociocultural contexts, there remains a critical need for region-specific investigations that deepen our understanding of its functional impact. In Colombia, limited empirical evidence exists regarding how ADHD influences neuropsychological and academic outcomes in school-aged children. Addressing this gap, the present study aimed to determine the effect of ADHD on neuropsychological and academic performance in a sample of Colombian children enrolled in primary and secondary education compared to a control group. More specifically, the research sought to answer the following question: Does attention-deficit/hyperactivity disorder (ADHD) significantly affect neuropsychological and academic performance in Colombian children when compared to their neurotypical peers? It was hypothesized that children diagnosed with ADHD would exhibit significantly lower performance across neuropsychological domains—particularly executive functioning, working memory, and attentional control—as well as in academic achievement relative to children without the disorder.

## 2. Materials and Methods

### 2.1. Type of Research

This study employed a quasi-experimental research design [39]. The independent variable was the presence or absence of ADHD, while the dependent variable was the performance of the children on various neuropsychological and academic tasks (Table 2). Internal validity was supported by establishing baseline equivalence between the ADHD and control groups through a matching procedure. The study adhered to the ethical principles outlined in the Declaration of Helsinki and received approval from the Institutional Scientific Ethics Committee at Universidad de Manizales, Colombia (ISET-03-22-0012) on 3 February 2022.

### 2.2. Sample

The final sample comprised 194 school-aged children from Manizales, evenly divided between those with a clinical diagnosis of ADHD and neurotypical controls. Groups were matched for sex, age, educational level, and socioeconomic strata (socioeconomic status) (Table 3). Each group included 24 girls and 73 boys, with ages ranging from 5 to 14 years (M = 9.4, SD = 2.7). Within the ADHD group, 59.8% met diagnostic criteria for the combined presentation, while the remainder exhibited predominantly inattentive symptoms.

### 2.3. Procedure

Data on the neuropsychological and academic assessments of children diagnosed with ADHD and control cases were collected over recent years as part of fieldwork conducted in various research projects. These projects were led by a Neuropsychopedagogy Specialization Program at a private university located in a central Colombian city within the Coffee Axis region. The first phase involved contacting school administrators in Manizales and presenting the research objectives. Schools expressing interest in participation were invited to schedule meetings with students’ families to extend invitations for study enrollment. Parents who demonstrated willingness to participate provided informed consent. The assessment process was conducted on school premises, where school administrators and parents authorized the participation of children in the study.

The sample selection process followed a purposive sampling strategy, relying on convenience sampling. An interdisciplinary team was responsible for determining group assignments, including professionals from various fields, such as medicine, psychiatry, psychology, and neuropsychology.

Ultimately, the study included 97 participants in the ADHD group and 97 in the control group. Both groups underwent neuropsychological and academic assessments using selected subtests from the ENI battery [40].

### 2.4. Inclusion Criteria

1. A minimum full-scale IQ score of 85, as measured by the abbreviated version of the Wechsler Intelligence Scale for Children (WISC-III), CX6 form (San Antonio, TX, USA) [41].
2. For group classification, children in the ADHD group were required to have T-scores ≥ 65 on the inattention and hyperactivity/impulsivity dimensions of the Conners Rating Scales (NY, USA), as reported by both parents and teachers. In contrast, children in the control group were required to have T-scores ≤ 50 for the same dimensions [9].
3. Provision of written informed consent by a parent or legal guardian, in accordance with institutional ethical standards.

### 2.5. Instruments

1. Screening to Determine Inclusion and Exclusion Criteria.
2. Conners’ Parent Rating Scale (CPRS) and Teacher Rating Scale (CTRS) [7]. Conners’ Rating Scale is a widely used behavioral assessment tool completed by parents and teachers to identify symptoms associated with ADHD. It is one of the most frequently employed instruments for evaluating behavioral difficulties in children within the Colombian context. The scale assesses a range of psychopathological domains commonly linked to ADHD, including hyperactivity, anxiety, depression, somatization, and behavioral and academic problems. A Spanish-language adaptation of the scale has been developed and validated, incorporating both factor analysis and normative data. For the present study, the version standardized for Colombian children was utilized to ensure cultural and contextual relevance.
3. WISC III [42], abbreviated form C6 x2: Vocabulary and Block Design Subscales [41]. This abbreviated version employs the vocabulary and block design subtests. This is a standardized clinical instrument designed to assess intellectual functioning in children aged 6 to 16 years and 11 months. It is administered individually and provides composite scores for verbal IQ, performance IQ, and full-scale IQ, offering reliable estimates of a child’s overall intellectual ability.
4. Semi-Structured Psychiatric Interview MINI-KID (Mini International Neuropsychiatric Interview for Children and Adolescents) [43]. This is a structured clinical diagnostic interview developed in accordance with DSM-IV and ICD-10 criteria. It is designed for individuals aged 6 to 17 years and 11 months and allows efficient administration, typically requiring approximately 25 min. Modeled after the adult version (MINI), the MINI-KID employs a branching logic based on key screening questions to determine the presence or absence of psychiatric disorders, thereby minimizing unnecessary items and administration time. The instrument assesses 23 psychiatric disorders, organized into modular sections, and includes a dedicated module for suicide risk assessment. Administration is preferably conducted in the presence of a parent or legal guardian, with items read verbatim to ensure standardization. Responses are binary (“yes” or “no”), and the progression through each module depends on initial screening responses. In the present study, the validated Spanish-language version of the MINI-KID was employed.
5. Academic and Neuropsychological Performance [40]. The ENI battery is a standardized instrument designed to assess neuropsychological development in Spanish-speaking children aged 5 to 16 years. The neuropsychological components of the ENI evaluated a broad range of cognitive functions, including visual and auditory attention (e.g., picture cancellation, letter cancellation, digit span in progression and regression), verbal and visual memory (e.g., word list recall, free and cued recall, auditory–verbal recognition, complex figure copy and recall, and delayed recall), cognitive flexibility, and language abilities (e.g., instruction following). In addition, the protocol assessed metalinguistic skills (e.g., synthesis, spelling, phoneme counting, and word counting) as well as verbal fluency across both semantic and phonemic categories. Academic abilities were evaluated through ENI subtests that measure reading (accuracy, comprehension, and speed) and writing (narrative composition, accuracy, and speed). The use of this battery provided a comprehensive evaluation of the participants’ neuropsychological and academic profiles within a culturally and linguistically appropriate framework.

### 2.6. Data Analysis

Based on the available data, a data matrix was constructed and subjected to the following statistical analysis using the Jamovi statistical package (V. 2.6.26, Sydney, Australia). This study described the variables using the mean and standard deviation. The Shapiro–Wilk test was used to assess the normality of the variables, while Levene’s test was applied to evaluate the homogeneity of variances. The experimental and control groups were compared using an independent-sample *t*-test for normally distributed data with homogeneous variances, Welch’s *t*-test for normally distributed data with non-homogeneous variances, or the Mann–Whitney *U* test for non-normally distributed data with non-homogeneous variances. Correlation analyses between variables, distinguishing between case and control groups, were conducted using Pearson’s correlation coefficient when the normality assumption was met, or Spearman’s correlation coefficient otherwise [44].

## 3. Results

### Description and Comparison Between the Experimental and Control Groups

Regarding the variables of interest in the present study (Table 4), the following findings were observed. The mean total intelligence quotient (IQ) was higher in the control group than in the ADHD group, with the latter also exhibiting greater homogeneity in IQ scores. Similarly, across all assessed metalinguistic skills, the control group had higher mean scores and greater homogeneity compared to the ADHD group, suggesting greater variability within the ADHD group in tasks such as synthesis, phoneme counting, spelling, and word counting. In terms of reading, the only measure in which the ADHD group exhibited a higher mean score was the number of words with errors in oral reading (NW: EO). For all other reading measures, the ADHD group had lower mean scores than the control group, with a lower coefficient of variation for NW: EO, indicating greater homogeneity in the number of errors within the ADHD group.

In writing, the ADHD group demonstrated higher mean scores in the number of errors in copying (NEW: C), the number of words with errors in written retrieval (NEW: WR), and writing retrieval speed (WRS). However, in all other writing measures, the ADHD group had lower mean scores than the control group (Table 4). Regarding memory, all assessed variables, except for visual memory/recall score of the complex figure (VMRSCF), had higher mean scores in the control group. Notably, for VMRSCF, the ADHD group displayed significant variability in scores (70% dispersion) compared to the control group (30%) (Table 5).

Attention-related measures indicated that the ADHD group had higher mean scores in commission of drawings (CD), omission of letters (OL), and total letter errors (TEL). Furthermore, the attention-related variables generally exhibited coefficients of variation of 150% or higher, indicating substantial heterogeneity in both groups (Table 5). The executive function (cognitive flexibility) measures revealed that the ADHD group had higher mean scores in the number of administered trials (CF:NAT), total errors (TE), percentage of errors (PE), number of perseverative responses (NPR), percentage of perseverative responses (PPR), and number of initial conceptualization trials (NICT) (Table 5).

In contrast, for all other executive function measures, the ADHD group had either lower or equal mean scores compared to the control group. Additionally, high coefficients of variation (40% or greater) were observed in most executive function measures, suggesting considerable heterogeneity within both groups. Lastly, in language-related assessments, the control group exhibited higher mean scores across all measured variables, while the ADHD group demonstrated greater variability in scores, as indicated by higher coefficients of variation (Table 6). These results highlight significant cognitive, linguistic, and attentional differences between children with ADHD and their neurotypical peers, with the former group generally exhibiting lower mean performance and greater variability across most tasks.

## 4. Discussion

The present study aimed to determine the effect of ADHD on neuropsychological and academic performance in a sample of Colombian children enrolled in primary and secondary education compared to a control group.

### 4.1. Sociodemographic Variables and ADHD

This study corroborates previous research indicating a higher prevalence of attention-deficit/hyperactivity disorder (ADHD) among boys compared to girls. However, the explanation for this difference appears more complex than previously assumed. Traditional research in the field has often attributed these gender disparities solely to neuroanatomical differences or variations in neurotransmitter functioning. For instance, some studies suggest that girls with ADHD exhibit a 10% reduction in gray matter volume compared to boys with ADHD. Additionally, girls are reported to reach peak cortical thickness approximately 3.5 years earlier than boys, suggesting distinct neurological developmental trajectories. Nevertheless, increasing evidence highlights the impact of social context and gender stereotypes on the timely diagnosis of ADHD in girls. Social norms often encourage girls to conform to expected behaviors, such as organization, obedience, dependence, and submission, potentially leading them to suppress disruptive behaviors to align with these societal expectations. As a result, girls may mask ADHD symptoms in the presence of caregivers and educators, complicating diagnosis and delaying intervention [43].

The higher prevalence of ADHD among boys observed in this study aligns with previous reports indicating male-to-female ratios of 4:1 or 3:1 [40,42,44,45]. More recent studies, however, suggest a lower ratio of approximately 2:1, where for every two or three diagnosed boys, one girl is identified with the disorder [46,47]. Traditionally, these gender differences were attributed to the greater frequency of ADHD diagnoses in boys [42,48], possibly due to the higher prevalence of the hyperactive–impulsive or combined presentation in boys compared to the predominantly inattentive type observed in girls.

It is essential to consider that parents, teachers, and peers who interact with children diagnosed with ADHD may exhibit greater tolerance toward inattentive behaviors—more common in girls—compared to the overt hyperactive–impulsive symptoms often displayed by boys. This normalization of inattentive symptoms may contribute to underdiagnosis in girls, limiting access to timely interventions. Some researchers suggest that females tend to exhibit symptoms such as distractibility, disorganization, and forgetfulness, which are perceived as less disruptive than the hyperactive–impulsive behaviors typically seen in males. Consequently, inattentive symptoms may be overlooked or deemed insufficiently severe to warrant a diagnosis [43].

In general, ADHD tends to be more conspicuous in boys, particularly those with hyperactive–impulsive or combined presentations. Symptoms associated with these presentations include motor restlessness, difficulty remaining seated, excessive talking, trouble waiting for turns, interruption of conversations, and intrusion into others’ affairs. Additionally, impulsivity in boys with ADHD has been linked to an increased risk of accidents and early engagement in risky behaviors, such as substance use, early sexual activity, and suicidal ideation. Conversely, in girls, the predominantly inattentive presentation may result in delayed diagnosis. Girls with ADHD are more likely to exhibit internalizing disorders, such as anxiety and depression, which can mask ADHD symptoms and further complicate diagnosis. Consequently, difficulties in following instructions, completing tasks, maintaining necessary resources, and frequent distractibility may go unnoticed [49].

Indeed, some have suggested that for girls to receive an ADHD diagnosis, their symptoms must be sufficiently pronounced or highly disruptive. Research indicates that females may require a higher symptom severity threshold to be diagnosed with ADHD. As a result, girls are often referred for psychiatric consultation only when inattention symptoms significantly impair their academic and social performance or lead to evident functional impairment [49,50]. In the present study, the mean age of children was 9 years, which is considered representative for this research. This finding is consistent with the diagnostic age range for children with ADHD as defined by the American Psychiatric Association [12], which places the diagnosis between the ages of 7 and 12. However, the present findings diverge from those reported in a study conducted by the National Survey of Children’s Health (NSCH), which found an average symptom onset age of approximately 6 years. Additionally, the NSCH study reported cases of severe ADHD being diagnosed even earlier, while milder cases were diagnosed approximately one year later [51].

Regarding the socioeconomic status of participants included in this research, despite the convenience sampling method, a higher percentage of children diagnosed with ADHD came from middle socioeconomic strata, which are equivalent to strata 3 and 4 in Colombia. Moreover, the majority of diagnosed children attended private schools in the city.

This finding contrasts with previous studies, which indicate a higher prevalence of ADHD in lower socioeconomic strata (strata 1 and 2 in Colombia). Most prior research describes associations between low socioeconomic status and factors such as social vulnerability, limited cultural and economic capital, low parental education (particularly maternal education), and a lack of opportunities—all of which are linked to a higher likelihood of ADHD diagnosis, initiation of medical treatment, and difficulties with classroom concentration and school adaptation [52,53,54].

Although the present study is limited by its convenience sampling method, the results suggest a trend toward increased awareness, knowledge, and education among middle-class families regarding the clinical and educational implications of ADHD. This awareness appears to facilitate timely diagnosis and intervention, allowing children with ADHD to better adapt to the high academic demands of modern society [55,56].

The fact that most children diagnosed with ADHD were enrolled in private schools and came from middle-income families suggests a prioritization of educational opportunities despite limited financial resources. This aligns with previous studies emphasizing the role of ADHD awareness in shaping societal norms and attitudes toward mental health issues (e.g., reducing stigma-related fears) and improving access to specialized educational support [49,56].

Among the sample, the most prevalent ADHD presentation was the combined type (59.8%), followed by the predominantly inattentive type (40.2%). Both figures exceed those reported in other studies, which estimate the prevalence of the combined type at 31.4% and the inattentive type at 32.2% in various global populations [10,11,12].

Classic studies have indicated that ADHD affects up to 1 in 20 children in the United States [57], with more recent prevalence estimates reaching 12.9% among American children [58]. Similarly, research in other countries has confirmed ADHD prevalence rates ranging between 5% and 12% when applying DSM diagnostic criteria [59,60]. Regarding Colombia, previous studies have reported an ADHD prevalence of 11.5%, with higher representation of the combined type (6.4%) and inattentive type (4.8%) compared to the hyperactive–impulsive type (0.3%) [61]. In contrast, more recent reports indicate a prevalence of 10.3% in Africa, with the inattentive type being most common (46.7%), followed by the hyperactive–impulsive type (33.7%) and the combined type (20.6%) [11].

In the present study, the predominance of the combined and inattentive types suggests increased recognition by families and teachers of the impact of inattention-related symptoms on diagnostic referrals and early ADHD identification. Traditionally, hyperactive–impulsive symptoms prompted more frequent reports from parents and teachers. However, attentional difficulties are now gaining recognition due to their association with long-term academic underachievement, lower overall academic performance, and higher dropout rates [62,63].

### 4.2. Intellectual Abilities and ADHD

Intelligence quotient (IQ) scores were higher among the control participants than among those diagnosed with ADHD. This result aligns with previous findings reporting lower general intellectual ability in ADHD cases compared to controls [64]. However, this finding contrasts with two recent studies. The first, conducted in China, evaluated 772 children aged 6 to 12 years with ADHD and found that their IQ scores fell within the neurotypical range. Additionally, no significant differences were observed in total IQ (TIQ), verbal IQ (VIQ), or performance IQ (PIQ) across ADHD subtypes [65]. The second study, conducted in Ecuador, assessed 50 children aged 5 to 16 years and reported average intellectual ability scores [66].

Beyond the results regarding the intellectual capacity of children with ADHD, which may be inconclusive and contribute to stigmatizing the difficulties that may arise in their intellectual profile, these findings are relevant insofar as the estimation of intellectual capacity is considered a predictor of academic performance potential [67]. Thus, it is important to assess the performance of children with ADHD in this measure to identify strengths and opportunities for implementing pedagogical, curricular, and didactic adaptations tailored to their intellectual profile. According to a study, this reinterpretation of intelligence scale analysis encourages the triangulation of information from other sources (teacher observations in class, performance tests, classroom innovations, etc.), aiming to enhance the understanding of clinical teams, teachers, and families regarding the student’s cognitive functioning [68]. This, in turn, fosters educational adjustments and adaptations that can be implemented both in school and at home [69].

### 4.3. Academic Performance and ADHD

Regarding the academic performance by the children with ADHD included in this study—specifically in metalinguistic skills, reading, and writing—in general, the findings reveal that the control group presented higher mean scores across most measures. The only tasks where the ADHD group obtained a higher mean score were those with evident clinical significance. These included reading tasks where the ADHD group exhibited a higher mean score for the number of reading errors in oral reading and writing tasks where they showed a higher mean number of errors in copying, written recall, and writing speed.

This result aligns with previous studies that have described reading difficulties related to decoding speed and text comprehension and difficulties in expressive vocabulary and word reading among children with ADHD [70,71,72]. Some research has even indicated that approximately 60% of children with reading disorders (RD) meet the criteria for at least one coexisting disorder. The most common of these in ADHD present in at least 20–40% of cases [73]. Similarly, previous studies have described the fact that texts by children and adolescents with ADHD, compared to control samples, do not necessarily differ in length but show difficulties in structure, coherence, and ideation related to concept formation. Additionally, spelling difficulties are frequently observed and are likely associated with the neuropsychological profile characteristic of children with ADHD. This profile includes challenges in attentional and executive functions, such as working memory, inhibitory control, set shifting, and sustained attention, which are considered predictors of reading and writing processes [74,75,76].

### 4.4. Neuropsychological Performance and ADHD

In this same vein, the neuropsychological profile assessment conducted in the present study considered attention, memory, executive functions, and language as essential cognitive processes in academic learning. Based on the results obtained by the ADHD group, the results indicate that the control group showed higher average scores in almost all tasks, while the ADHD group consistently exhibited lower scores. More specifically, the ADHD group had lower mean scores compared to the control group, except in the visual memory task (complex figure recall score), where the ADHD group performed better. Conversely, the ADHD group exhibited higher scores—indicating greater difficulties—in attentional variables, such as commission errors in drawings, omission of letters, and total letter errors.

Regarding executive functions, various difficulties related to cognitive flexibility were evident. The ADHD group showed higher mean scores, relative to controls, in clinically significant variables that indicate challenges in the number of trials administered, total errors, percentage of errors, number of perseverative responses, percentage of perseverative responses, and the number of initial conceptualization trials. In terms of language, the control group exhibited higher mean scores across all measures compared to the ADHD group.

Overall, the findings of this study support previously described neuropsychological difficulties in children with ADHD, including deficits in selective and sustained attention, working memory, and long-term memory. Executive function challenges were also observed, particularly in cognitive flexibility, the ability to integrate environmental feedback, and behavioral regulation. Additionally, difficulties in verbal fluency—both phonological and semantic—were identified, affecting the production of words that start with a specific letter or phoneme and those belonging to a given semantic category. Finally, the results are consistent with previous studies that have reported difficulties in following instructions and metalinguistic skills.

These findings align with research that has characterized the cognitive profile of children with ADHD as featuring overall lower executive function and academic ability scores, although still within the normal range based on cultural benchmarks. However, these lower scores are significantly below those of control groups and affect academic functionality, learning potential, and life skills [28,77]. These difficulties appear to be exacerbated by the low performance of children with ADHD in certain cognitive functions considered prerequisites for academic skills, such as working memory, processing speed, and attention [78].

An important aspect of this study is the heterogeneity and dispersion of scores within the ADHD group in the evaluation of certain cognitive processes, particularly metalinguistic skills, memory, attention, and executive functions. This result appears to confirm the heterogeneity in the clinical manifestation of ADHD, a topic extensively addressed in recent research identifying novel ADHD profiles [18,26].

Among these studies, one notable investigation involved 854 ethnically diverse adolescents aged 10 to 17 years, in which cognitive profiles were assessed to determine whether they differed based on individual characteristics such as age, gender, race, and level of family adversity. The study identified new ADHD profiles: (1) Simple ADHD (63.7%), characterized by a mix of inattentive and combined ADHD subtypes, moderate levels of impairment, and infrequent comorbidities; (2) ADHD + Internalizing (11.4%), marked by a higher likelihood of comorbid anxiety and/or depression; and (3) Disruptive/Disorganized ADHD (24.9%), characterized by severe problems in organization, time management, and planning (OTP), which was also the combined ADHD subtype frequently exhibiting disruptive behavior at school [18]. Recognizing the intellectual, cognitive, and academic profile characteristics of children with ADHD has become even more crucial in the post-pandemic years. The challenges emerging from the numerous educational changes and curricular adjustments prompted by virtual learning experiences highlight the need for greater intervention efforts. These efforts should address the heterogeneity of the disorder and implement more differentiated and personalized interventions targeting executive functions essential for self-regulated learning. In students with ADHD diagnoses who struggle with working memory and attention, these difficulties may further limit their ability to manage their own learning process.

## 5. Limitations and Future Research Directions

One of the primary limitations of this study lies in the use of a convenience sampling method. This approach inherently limits the generalizability of findings, as not all segments of the target population have an equal probability of being included, and thus, the sample may not fully reflect the broader population’s diversity. Nevertheless, the data were collected using well-calibrated protocols and culturally adapted normative standards, and participants were selected based on strict clinical criteria. These factors enhance the interpretability and applicability of the findings to similar sociocultural contexts.

The objective assessment of ADHD effects on cognitive and academic variables requires the use of standardized, individually administered tasks that incorporate contextually valid norms and allow comparisons between clinical and normative reference groups. However, the comprehensive nature of these neuropsychological and academic assessments often necessitates lengthy protocols. As a result, despite the ambitious scope of this study, it was not feasible to include all variables of interest. Notably, academic domains such as mathematical learning—encompassing arithmetic operations, memorization of facts, fluency, and mathematical reasoning—remain underexplored in the context of ADHD and warrant further investigation. Similarly, the present study did not assess the potential impact of ADHD on emotional and affective development, despite their relevance. This omission was necessary to maintain a feasible assessment burden for participating children and their families.

Future research should address ADHD from a mixed-methods perspective, integrating qualitative insights from children and families with quantitative data to enrich the understanding of ADHD’s impact. Furthermore, it is essential to advance research within the Colombian and broader Ibero-American contexts by examining functional impairment associated with ADHD and its long-term correlation with academic performance, particularly during higher education. These efforts would contribute meaningfully to the development of culturally informed diagnostic frameworks and intervention strategies.

## 6. Conclusions

This study aimed to examine the effect of ADHD on the neuropsychological and academic performance of a sample of Colombian children in primary and secondary education compared to a control group. The findings revealed that children with ADHD exhibited lower cognitive, linguistic, and attentional performance, as well as greater variability in these domains relative to their neurotypical peers. Deficits were particularly pronounced in metalinguistic abilities, reading, writing, memory, attention, and executive functioning. Given the clinical relevance of ADHD and its significant impact on academic achievement, further research is warranted—particularly studies that incorporate underexplored variables such as mathematical learning, as well as the influence of emotional functioning, motivational factors, parenting styles, and instructional approaches. These dimensions are essential to better understand adaptive functioning in school and family environments. Children with ADHD possess significant potential; however, greater awareness and contextual support are needed to provide them with the appropriate educational and psychosocial interventions to overcome their challenges.

## Figures and Tables

**Table 2:** Quantitative variables.

Variable Group	Variable	Variable Abbrev.
Writing	Writing Accuracy: Syllable Dictation	WA: SD
	Writing Accuracy: Word Dictation	WA: WD
	Writing Accuracy: Non-Word dictation	WA: NWD
	Writing Accuracy: Sentence Dictation	WA: SD
	Number of Words with Errors in Copying	NWE:C
	Number of Words with Errors in Written Retrieval	NWE: WR
	Narrative Composition Analysis: Narrative Coherence	NCA: NC
	Narrative Composition Analysis: Written Retrieval Length	NCA: WRL
	Copying Speed	CS
	Written Retrieval Speed	WRS
Reading	Reading Accuracy: Syllables	RA: S
	Reading Accuracy: Words	RA:W
	Reading Accuracy: Non-Words	RA: NW
	Reading Accuracy: Sentence Reading	RA: SR
	Number of Words with Errors in Oral Reading	NW: EO
	Reading Comprehension: Sentence Reading	RC: SR
	Reading Comprehension: Oral Reading	RC: OR
	Reading Comprehension: Inferential Response in Oral reading (item 4)	RC: IROR4
	Reading Comprehension: Silent Reading of a Text	RC: SRT
	Reading Speed	RS
	Silent Reading Speed	SRS
Intelligence Quotient	Verbal Intellectual Quotient Measure—Vocabulary Task	VIQ-Voc
	Performance IQ Assessment—Block Design Task	PIQA-BD
	Full-Scale IQ score	FS: IQS
Memory	Visual memory: copy score of the complex figure	VM: CSCF
	Visual memory: recall score of the complex figure.	VM: RSCF
	Coding: Word List	C: WL
	Coding/Word List/Working Memory—First Trial	CWL-WM- FT
	Coding Spontaneous Recall	CDR
	Coding Delayed Recall with Cues	CDRC
	Verbal Auditory Recognition	VAR
Attention	Visual Attention—Drawing Cancellation Task	VA: DCT
	Omission of Drawings	OD
	Commission of Drawings	CD
	Visual Attention: Letter Cancellation Task	VA: LCT
	Omissions: Letters	OL
	Commissions: Letters	CL
	Total Errors: Letters	TEL
	Auditory Attention: Forward Digit Span Task	AA: FDS
	Auditory Attention: Backward Digit Span Task	AA: BDS
Executive Functioning	Cognitive Flexibility: Number of Administered Trials	CF: NAT
	Cognitive Flexibility: Total Correct Responses	CF: TCR
	Cognitive Flexibility: Total Errors	CF: TE
	Cognitive Flexibility: Percentage of Errors	CF: PE
	Cognitive Flexibility: Number of Categories	CF: NC
	Cognitive Flexibility: Inability to Maintain Set	CF: IMS
	Cognitive Flexibility: Number of Perseverative Responses	CF: NPR
	Cognitive Flexibility: Percentage of Perseverative Responses	CF: PPR
	Cognitive Flexibility: Number of Initial Conceptualization Trials	CF: NICT
	Semantic Verbal Fluency (Animals)	SVF: A
	Phonemic Verbal Fluency (Letter M)	PVF: M
Language	Instruction Following Task	IFT
	Metalinguistic skills: Synthesis task	MS: ST
	Metalinguistic Skills: Sound Counting Task	MS: SC
	Metalinguistic Skills: Spelling Task	MS: SPT
	Metalinguistic Skills: Word Counting Task	MS: WCT

Note: All cognitive and academic measures in this study were assessed using subtests from the Child Neuropsychological Assessment Battery (ENI) (Mexico, Mexico). Administration procedures adhered strictly to the standardized guidelines established by the test developers, as the ENI is considered the gold standard for pediatric neuropsychological assessment in Ibero-America and includes normative data specific to the Colombian population. Due to the length and specificity of each subtest, readers are encouraged to consult the ENI manual for a detailed description of the individual tasks.

**Table 3:** Sociodemographic variables.

Variable	Descriptive Statistics	Case	Control
Age	Mean	9.41	9.43
	Standard Deviation	2.7	2.67
	Coefficient of Variation	28.6%	28.3%
Sex	Female %	24.7	24.7
	Male %	75.3	7.53
Socioeconomic status	Strata 1%	19.6	19.6
	Strata 2%	67.0	67.0
	Strata 3%	13.4	13.4
	Strata 6%	1.0	1.0
Education level	Preschool %	5.2	3.1
	First Grade%	16.5	15.5
	Second Grade %	14.4	12.4
	Third Grade %	14.4	16.5
	Fourth Grade %	6.2	9.3
	Fifth Grade %	12.4	7.2
	Sixth Grade %	11.3	12.4
	Seventh Grade %	12.4	10.3
	Eight Grade %	4.1	7.2
	Ninth Grade %	3.1	5.2
	Tenth Grade %	0.0	1.0

**Table 4:** Statistics for writing and reading variables.

Variable	Case (Mean ± SD)	Control (Mean ± SD)	*p*-Value	Effect Size (*d*) (R-BC)
Writing				
WA: SD	6.134 ± 2.519	7.0208 ± 1.717	**0.005**	0.41140 (*d*)
WA: WD	3.969 ± 1.95	5.0833 ± 1.89	**<0.001**	0.58033 (*d*)
WA: NWD	4.814 ± 2.078	5.5104 ± 1.536	**0.036**	0.16527 (R-BC)
WA: SeD	9.474 ± 5.803	11.5 ± 5.872	**0.017**	0.34710 (*d*)
NWE: C	9.329 ± 7.486	6.4719 ± 6.01	**0.006**	0.42098 (*d*)
NWE: WR	14.783 ± 10.001	12.4889 ± 7.581	0.284	0.09438 (R-BC)
NCA: NC	3.667 ± 1.616	4 ± 1.773	0.194	0.19653 (*d*)
NCA: WRL	70.56 ± 44.453	79.0652 ± 45.453	0.211	0.18920 (*d*)
CS	11.202 ± 6.182	12.4945 ± 6.72	0.187	0.20012 (*d*)
WRS	14.718 ± 9.154	13.5543 ± 7.425	0.360	0.13963 (*d*)
Reading				
RA: S	6.505 ± 2.658	7.3542 ± 1.735	**0.017**	0.16323 (R-BC)
RA: W	9.526 ± 3.028	10.4271 ± 1.868	**0.016**	0.15174 (R-BC)
RA: NW	6.052 ± 2.252	6.8646 ± 1.626	**0.003**	0.23647 (R-BC)
RA: SR	7.758 ± 3.231	8.9583 ± 2.166	**0.002**	0.23673 (R-BC)
NW: EO	4.694 ± 4.952	2.4409 ± 3.002	**<0.001**	0.38558 (R-BC)
RC: SR	6.295 ± 2.82	7.0313 ± 2.305	0.050	0.28593 (*d*)
RC: OR	4.539 ± 2.468	5.5789 ± 1.998	**0.003**	0.24802 (R-BC)
RC: IROR4	1.056 ± 0.774	1.2935 ± 0.719	**0.034**	0.31761 (*d*)
RC: SRT	3.512 ± 2.034	4 ± 2.047	0.119	0.23936 (*d*)
RS	74.141 ± 42.451	92.2553 ± 46.433	**0.007**	0.40718 (*d*)
SRS	79.988 ± 49.172	97.0238 ± 50.899	**0.030**	0.34043 (*d*)

Note: WA: SD = Writing Accuracy: Syllable Dictation, WA: WD = Writing Accuracy: Word Dictation, WA: NWD = Writing Accuracy: Non-Word Dictation, WA: SeD = Writing Accuracy: Sentence Dictation, NWE: C = Number of Words with Errors in Copying, NWE: WR = Number of Words with Errors in Written Retrieval, NCA: NC = Narrative Coherence Accuracy, NCA: WRL = Written Retrieval Length, CS = Copying Speed, WRS = Written Retrieval Speed, RA: S = Reading Accuracy: Syllable, RA: W = Reading Accuracy: Words, RA: NW = Reading Accuracy: Non-Words, RA: SR = Reading Accuracy: Sentence Reading, NW: EO = Number of Words with Errors in Oral Reading, RC: SR = Reading Comprehension: Sentence Reading, RC: OR = Reading Comprehension: Oral Reading, RC: IROR4 = Reading Comprehension: Inferential Response in Oral Reading, RC: SRT = Reading Comprehension: Silent Reading of a Text, RS = Reading Speed, SRS = Silent Reading Speed.

**Table 5:** Statistical for intelligence quotient, memory, attention, executive functioning variables.

Variable	Case (Mean ± SD)	Control (Mean ± SD)	*p*-Value	Effect Size (*d*) (R-BC)
Intelligence Quotient				
VIQ: Voc	25.24 ± 8.142	29.6354 ± 9.418	**<0.001**	0.499 (*d*)
PIQA-BD	28.063 ± 14.633	32.1146 ± 14.294	0.054	0.280 (*d*)
FS: IQS	19.742 ± 4.391	23.0313 ± 5.387	**<0.001**	0.34912 (R-BC)
Memory				
VM: CSCF	7.835 ± 2.741	7.9063 ± 2.726	0.857	0.026 (*d*)
VM: RSCF	86.557 ± 34.62	90.875 ± 42.74	0.442	0.111 (*d*)
C: WL	26.155 ± 7.742	28.25 ± 7.182	**0.032**	0.178 (R-BC)
CWL-WM- FT	4.763 ± 1.841	5.5313 ± 1.515	**0.002**	0.455 (*d*)
CSR	7.237 ± 2.188	7.7708 ± 2.382	0.107	0.233 (*d*)
CDRC	7.278 ± 2.035	7.8333 ± 2.378	0.083	0.250 (*d*)
VAR	19.443 ± 3.416	19.7917 ± 4.203	0.528	0.090 (*d*)
Attention				
VA: DCT	19.835 ± 9.077	20.3646 ± 9.874	0.699	0.055 (*d*)
OD	2.454 ± 4.1	2.7604 ± 5.761	0.671	0.061 (*d*)
CD	0.742 ± 1.856	0.5 ± 1.306	0.295	0.151 (*d*)
VA: LCT	22.804 ± 10.429	24.8854 ± 11.981	0.200	0.18531 (*d*)
OL	3.608 ± 7.927	1.5625 ± 2.854	**0.003**	0.23711 (R-BC)
CL	0.443 ± 0.968	0.3854 ± 1.268	0.722	0.05132 (*d*)
TEL	4.052 ± 7.97	1.9479 ± 3.014	**0.003**	0.23872 (R-BC)
AA: FDS	4.907 ± 1.001	5.0208 ± 1.248	0.487	0.10044 (*d*)
AA-BDST	3.206 ± 1.04	3.5521 ± 1.23	**0.036**	0.30366 (*d*)
Executive Functioning				
CF: NAT	50.25 ± 6.383	50.1458 ± 6.066	0.908	0.01673 (*d*)
CF: TCA	33.667 ± 6.609	34.7604 ± 5.783	0.224	0.17613 (*d*)
CF: TE	16.412 ± 9.273	15.3854 ± 8.231	0.417	0.11713 (*d*)
CF: PE	31.708 ± 16.016	29.5313 ± 14.184	0.320	0.14392 (*d*)
CF: NC	1.927 ± 0.965	2.0625 ± 0.938	0.326	0.14228 (*d*)
CF: IMS	0.615 ± 0.8	0.6458 ± 0.821	0.790	0.03857 (*d*)
CF: NPR	12.063 ± 11.312	9.5625 ± 8.257	0.273	0.09147 (R-BC)
CF: PPR	22.969 ± 20.673	18.3958 ± 14.97	0.281	0.09006 (R-BC)
CF: NICT	16 ± 10.009	14.8438 ± 9.476	0.412	0.11863 (*d*)
SVF: A	14.216 ± 5.134	15 ± 5.07	0.287	0.15356 (*d*)
PVF: M	5.619 ± 3.67	6.2708 ± 3.782	0.226	0.17504 (*d*)

Note: VIQ: Voc = Verbal Intellectual Quotient: Vocabulary Task, PIQA-BD = Performance Intellectual Quotient Assessment—Block Design, FS: IQS = Full-Scale Intellectual Quotient, VM: CSCF = Visual Memory: Copy Score of the Complex Figure, VM: RSCF = Visual Memory: Recall Score of the Complex Figure, C: WL = Coding: Word List, CWL-WM-FT = Coding Word List–Working Memory—First Trial, CSR = Coding Spontaneous Recall, CDRC = Coding Delayed Recall with Cues, VAR = Verbal Auditory Recognition, VA: DCT = Visual Attention: Drawing Cancellation Task, OD = Omission of Drawings, CD = Commission of Drawings, VA: LCT = Visual Attention: Letter Cancellation Task, OL = Omission of Letters, CL = Commission of Letters, TEL = Total of Errors in Letters, AA: FDS = Auditory Attention: Forward Digit Span, AA-BDST = Auditory Attention—Backward Digit Span task, CF: NAT = Cognitive Flexibility: Number of Administered Trials, CF: TCA = Cognitive Flexibility: Total Correct Answers, CF: TE = Cognitive Flexibility: Total Errors, CF: PE = Cognitive Flexibility: Percentage of Errors, CF: NC = Cognitive Flexibility: Number of Categories, CF: IMS = Cognitive Flexibility: Inability to Maintain Set, CF: NPR = Cognitive Flexibility: Number of Perseverative Responses, CF: PPR = Cognitive Flexibility: Percentage of Perseverative Responses, CF: NICT = Cognitive Flexibility: Number of Initial Conceptualization Trials, SVF: A = Semantic Verbal Fluency: Animals, PVF: M = Phonemic Verbal Fluency: Letter M.

**Table 6:** Statistics for language variables.

Variable	Case (Mean ± SD)	Control (Mean ± SD)	*p*-Value	Effect Size (*d*) (R-BC)
Language				
IFT	8.546 ± 1.458	9.0521 ± 1.251	**0.009**	0.20758 (R-BC)
MS: ST	2.557 ± 2.194	3.2917 ± 2.161	**0.020**	0.33751 (*d*)
MS: SC	4.381 ± 2.687	5.3958 ± 2.306	**0.008**	0.21886 (R-BC)
MS: SPT	4.144 ± 2.332	4.8229 ± 1.908	0.081	0.14412 (R-BC)
MS: WCT	3.711 ± 2.872	4.8646 ± 2.382	**0.006**	0.22444 (R-BC)

Note: IFT = Instruction Following Task, MS: ST = Metalinguistic Skills: Synthesis Task, MS: SC = Metalinguistic Skills Sound Counting Task, MS: SPT = Metalinguistic Skills Spelling Task, MS: WCT = Metalinguistic Skills Word Counting Task.

## Data Availability

The data presented in this study are available on request from the corresponding author. The data are not publicly available due to ethical standards.
